# Yellow Jessamine

**Published:** 1853-11

**Authors:** 


### *On the Therapeutic Properties of Yellow Jessamine.*

BY DR. BATCHELOR.

The yellow jessamine, (*Bignonia sempervirens, of Linnæus; Gelseminum sempervirens, of Jussieu,*) possesses undoubted remedial properties in a large class of diseases.

It has been used in neuralgia, rheumatism, intermittent, remittent and typhoid fevers, in pleurisy, pneumonia, in chorea and epilepsy, and in many cases with excellent results.

The gelseminum has a slight narcotic odour, and possesses narcotic, nervine, anti-spasmodic and sedative powers. In full doses it produces a sort of intoxication, languor, dizziness, double-vision, inability to raise the eye-lids; and in over-dose, complete muscular prostration. As a sedative, I consider it superior to digitalis, sanguinaria, or veratrum viride; it possesses the advantage of being by no means unpleasant to the taste, and of not producing nausea.

A nostrnm called the "electric febrifuge" is prepared from this article, distinguished by essence of wintergreen.

I prepared the medicine in the following manner: A bottle is loosely filled with the bark of the fresh root; equal parts of whisky and water are added, and the bark is mascerated for fourteen days. Twenty to sixty drops of this infusion may be used at a dose, alone or combined with quinine.—(*Trans. of Ala. Med. Assoc.*)

The "Tincture of Yellow Jessamine," Gelseminum," as it is generally called, forms the basis of several Patent Fever Remedies, much in vogue in the South-Western States. Its power as a febrifuge was discovered by accident. It is said that a negro was sent to dig for some well known root much used is Fever and Ague. *By mistake* he dug the root of Yellow Jessamine. Its effects thus becoming known, it passed through the usual course of every valuable remedy, being seized upon, prepared and sold as a patent medicine in various forms. One of these is the "Mississippi River Tonic."

The Thompsonians, (or Eclectics, as they now call themselves,) make great use of it. Hence the great demand for it in Cincinnati, where their principal school is situated.

Many physicians in regular practice employ it extensively, and speak highly of its effects. As stated above, it may be used alone or with quinine. EDITORS.

